# Increasing Mental Health Literacy in Law Enforcement to Improve Best Practices in Policing—Introduction of an Empirically Derived, Modular, Differentiated, and End-User Driven Training Design

**DOI:** 10.3389/fpsyt.2021.706587

**Published:** 2021-08-02

**Authors:** Katharina Lorey, Jörg M. Fegert

**Affiliations:** ^1^Ministry of the Interior, Digitalisation and Local Government of Baden-Wuerttemberg, Stuttgart, Germany; ^2^Child and Adolescent Psychiatry and Psychotherapy, Ulm University Medical Center, Ulm, Germany

**Keywords:** persons with mental disorders, law enforcement, best practice, training program design, survey, police, police officer

## Abstract

**Objective:** Law enforcement officers often have contact to persons who show symptoms of mental disorders. Adequately designed training is necessary for developing the best possible practices in policing when coming into contact with mentally ill people, and may help to expand their general knowledge on mental disorders. To achieve a sustainable implementation of training content in daily policing work, the acceptance and proactive integration of methods by the training participants is essential.

**Method:** This study investigates an exemplary modular training curriculum based on a survey with 2,228 German police officers (28.2% female, 71.8% male) concerning their needs and challenges when coming into contact with persons with mental disorders. This empirical end-user driven approach was used to adapt existing training concepts to the current needs and interests of law enforcement personnel in order to maximize compliance.

**Results:** The training program draft includes basic modules which are intended to be of direct interest to all police officers, such as mental disorders with high policing relevance, encountering suicidal patients, (non-directive) communication and de-escalation skills, and mental hygiene in policing. They are arranged in more specialized modules that address specific target group audiences within police forces and the training curriculum provides information about genuine risks and self-protection, trauma sensitivity, and interaction with children and victims among other contents. The self-selectable, modular, and empirically-based continued training program also includes an introduction to local mental health service professionals and networks, trialogue sequences, and situational role play scenarios.

**Conclusion:** Due to frequent contact law enforcement officers have to mentally ill people, improved training designed to maximize knowledge and the integration of trained methods is necessary. Gaining acceptance and proactive support by trainees is ensured through end-user driven implementation of specialized and differentiated up-to-date training programs. Our results showcase how police officers' perspectives on persons with mental illnesses is a main aspect that can and should be used to encourage training course designs.

## Introduction

Multiple studies suggest the necessity of adequate training of law enforcement officers to improve their handling of the frequent contact to mentally ill people (1–4). Police officers are indeed willing to learn about this topic and receive additional training (5, 6). We designed a training approach with the aim to improve the interaction between police and person with mental illnesses (PMI) on a qualitative level by surveying police officers' needs and challenges when encountering PMI. Based on these survey results,^1^ we developed an exemplary training curriculum with specific suggestions for relevant modules and contents.

In 1987, the 27-year-old Joseph Dewayne Robinson who was known to be mentally ill was shot multiple times by police officers in Memphis Tennessee. This incident caused the development of the Crisis Intervention Teams (CIT), the most common training courses within the United States as well as the theoretical background that dates back more than 30 years. Three decades of training thousands of police officers within the United States and internationally (e.g., Canada, United Kingdom, and Australia), have resulted in the dissemination of 2,700 CIT programs in the United States alone (7). Since its establishment and up until today the main goals of CIT focus on the quality of collaboration between law enforcement, mental health providers, hospital emergency departments, and individuals with mental illness and their families (8). However, most well-established training concepts have been developed decades ago and need to be updated using recent advancements in research on policing PMI.

Over the last years, new research has broadened the scientific view on mental health issues. For example, modern research approaches the correlation between violence and mental illness from a much more differentiated perspective and puts more emphasis on the importance of contextual factors to explain a potential connection (9). For police officers, such a differentiated approach can be crucial. When a mentally ill person is not expected to be automatically violent and aggressive, responding behavior may be adjusted and conflicts with potential lethal outcomes could be avoided. New programs such as, “the R-Model” crisis intervention de-escalation training (10), or the “Training and Education about Mental Illness for Police Organizations”—TEMPO (11) are in need of integrating the perspectives of their main target groups on challenges and needs, which arise from contacts with persons with mental illnesses: the law enforcement personnel itself (1, 12, 13). CIT programs have been developed especially as a police-based approach to provide first responders to emergency calls with methods to prevent inadequate decision-making, regarding arrest or custody involving a PMI. Furthermore, several programs will shortly be introduced.

Within our approach to this issue, maximizing the acceptance and ensuring the feasibility of training program contents in the eyes of trainees were two main pre-conditions, which we addressed by considering the target group's own training needs and knowledge gaps. Several studies have already pointed out the perspectives of police officers on their interactions with PMI (6, 14–16), as well as the aspects of frequencies (6), perceived danger and unpredictability (15, 17), and the consequences of lacking mental health literacy or communication skills (18, 19). Taking this into account, Thomas and Watson (3) suggest: “Training should be practical, applied and reinforced.” In our opinion, this paradigm should guide empirically-based approaches to training designs. Hence, the presentation of an example of how practical an applied and reinforced training course could look like in Germany, and potentially in other countries.

According to a study by the Treatment Advocacy Center (20) in the United States for example, people with untreated mental illness are 16 times more likely to be killed during a police encounter than other civilians approached or stopped by law enforcement. In addition, the issue received an increasing amount of public and political interest because of lacking mental health services and fatal outcomes of interactions between police and persons with mental illnesses. Addressing this discourse, New York City for example announced that they would initiate a trial program that aims to establish mental crisis response teams outside of the police force in some parts of the city in April 2021 (21), which signifies the strong demand for adequate public services in this field. The killing of Daniel Prude on March 23, 2020 in Rochester, New York, for example caused widespread outcries and significant criticism directed against the authorities. The fatal outcome of the police encounter in this case called for changes in how law enforcement personnel respond to PMI. This development indicates the pressing need to design updated training courses for police officers reflecting the current scientific understanding of mental illnesses and adequate responses to them. On the other hand, today's challenges and changes in policing work, as well as the significantly increased demands placed on officers in their daily work must be reflected in those trainings as well [e.g., (22)]. Such developments characterize the climate of law enforcement not just in the United States but in many other countries as well.

The presented end-user driven exemplary training course design includes the formulated needs of 2,228 German police officers we previously surveyed. Our main goal for the present study is to provide useful insights for training course development beyond national contexts and potentially relevant modules for policing requirements similar to Germany; where no standardized training on this issue exists for police officers. After several incidents with a fatal outcome involving mentally ill persons, some German states (e.g., Hamburg and Berlin) began to introduce training courses for officers. Whereas, police officers in Hamburg practice encounters with psychotic and bipolar patients in trialogical approaches (23). Berlin's police officers have the option to participate in a 4-day seminar that includes training sequences, law education and introduces network partners (24).

Research addressing the evidence of the effectiveness of training programs for police officers must be expanded. In this context, the question of how to define effectiveness (e.g., decreasing numbers of injuries or death and/or the number of successfully admitted patients to a psychiatric facility instead of jail) is controversially discussed. CIT's positive effects mostly focus on police officers' attitudes (e.g., reduced stigmatization), knowledge officer-level outcomes (e.g., officer satisfaction) and increased verbal negotiation skills instead of arresting (7, 25). These effects correspond with the evaluation of the effectiveness of German training programs, which first and foremost aim to reduce the stigmatization of PMI and decrease police officers use of force (23). Without disregarding the perspective of PMI themselves (26), experts of police psychology and psychiatry such as ourselves who led this project, suggest that this training design could serve as an example and invitation to incorporate the already fruitful scientific discourse and bi-directional research experiences between mental health and law enforcement professionals. Hence, in future research and training program design, those experts who are involved should be encouraged to address the question of effectiveness assessment at an early stage.

## Summarizing Existing Training Designs

### The CIT Model

Arguably among the best-known concepts is the CIT Model that was implemented in Memphis, Tennessee in 1988 which consists of a 40-h course. Originally, it incorporated mental health professionals, law enforcement officers, local advocacy groups, and the National Alliance on Mental Illness (NAMI). CIT addresses signs and symptoms of mental illness, such as Schizophrenia and psychotic disorders, affective disorders, cognitive disorders, addiction disorders, and anxiety disorders. Other components teach inter alia community resources and perspectives, communication techniques, needs of mental health consumers, and involuntary treatment. Didactical methods like role playing scenarios, audiovisual vignettes and de-escalation techniques are included, as well as field trips to local jails and psychiatric facilities.

Several modifications of the original CIT model emerged over the last couple of decades,^2^ typically accounting for differences in the locally available services or size of police departments. Nevertheless, the core elements of CIT usually combine the networking of law enforcement, community, research, and mental health professions, as well as include policing procedures and mental health facilities' availability.

### The R-Model

Between 2017 and 2018, the “Research-Response-Refer Model” was developed as a response to the changing climate of policing in the United States and included current knowledge about mental disorders, accessibility, cost-effectivity, and agency-specification. This consists of an 8-h in-house R-Model training, a problem overview, recognizable signs of crises and mental illness, management tools such as de-escalation and suicide prevention, aspects of trauma, treatment and resources were integrated, in addition to specific agencies' initiatives. It is worth noting that this training course design also encourages the invitation of guest speakers who suffer from serious mental illness themselves.

### TEMPO

The main scope of this training model is to educate all types of police officers on the broader societal dynamics behind modern policing philosophies, stigmatization, the judicial perspective on mental health issues, ethics, and attitude formation. Hence, the components of this course design mainly cover knowledge and skills related to understanding the nature and effects of mental illnesses, as well as the specific local environment in which the interaction between police and PMI occurs. Furthermore, intervention strategies, risk assessment, use of discretion, ethical decision making, self-evaluation and assessment are included. In particular, TEMPO makes a distinction between specific target group audiences (e.g., “front desk” personnel, victim service workers, crisis negotiators, ERT/SWAT commanders), learning level varieties (in sum five levels, TEMPO 100–500^3^), different time frames (e.g., TEMPO 101 is a 4-day module) and intensity. According to the authors of TEMPO, recommended methods in facilitating the learning effects include role playing scenarios, visiting sites of interest, testimonials of people affected by mental health issues, and simulation exercises. TEMPO also encourages the use of e-learning modules but leaves the specific implementation and method selection to be decided by each police organization (30).

### T3^TM^—Tact, Tactics, and Trust Training Program

In order to improve social interaction skills of United States soldiers in Iraq and Afghanistan, the Defense Advanced Research Project Agency (DARPA) developed the T3^TM^ model. McLean et al. (31) examined its effectiveness on police officers' attitudes and behaviors when encountering citizens after their skills in decision making, de-escalation, empathy, rapport building, and self-control were enhanced through this course. The training is based on several video sequences depicting actual police-citizen interactions, which are subsequently discussed and embedded in half- and full-day training sessions that aim to introduce additional concepts. T3^TM^ is not exclusively focused on police officers' contact with PMI.

### Mapping the Status Quo of Training Courses

According to a national survey among police departments, three basic training models can be distinguished within the United States (32): police-based specialized mental health response, such as Community Service Officers (CSO, see also *Birmingham's CSO Program*), police-based specialized law enforcement response (e.g., CIT program), and specialized mental health responses situated within the healthcare system itself, such as the mobile crisis unit (MCU, see also *The Knoxville mobile mental health crisis unit*). The Birmingham CSO Program can briefly be described as an in-house support mechanism for crisis intervention and follow-up assistance. Therefore, civilian dressed, unarmed community service officers employed by the police department without the authority to arrest, collaborate with police officers in cases of mental health emergencies. After participating in a 6-week training sequence and gathering field experiences, the CSOs are available in those urban police precincts which respond to mental health emergencies and attend to various additional forms of social service or other requests for general assistance. The so-called mobile crisis units (MCU) are comprised of community mental-healthcare-based crisis teams and interact with police departments in communities that have established a close liaison between law enforcement and mental health professionals. The MCUs typically respond to calls without a necessity for hospitalization or arrest, such as community support requests, or calls for assistance from jails and emergency rooms.

Furthermore, Community-oriented policing (COP) (which does not have a dedicated focus on interaction with PMI) addresses the relationships between law enforcement personnel and community members. Similar to problem-oriented policing (POP) as a preventive approach and most of the previously mapped concepts, their general contribution can be defined as enhancing officers' problem-solving skills.

In addition, Greenstone (33) and Shinder (34) refer to the similarities between two special types of law enforcement personnel, which are both confronted with crisis situations on a regular basis: police negotiators and crisis intervention units. Since the lead author of this study possesses extensive personal experience regarding the psychological training of police negotiators within the German police, we also chose to include this particular training perspective here. Indeed, situations which require specially trained negotiators, such as suicide (attempts), domestic violence, active threat scenarios, and hostage situations are frequently linked to symptoms of (temporary or chronical) mental disorders. Hence, exploring the training of police negotiators in regard to potentially valuable and transferable lessons for the general law enforcement community appears warranted. The training of police negotiators that was previously mentioned, which was conducted by the lead author of this study was repeated over a course of 9 years and each training was 3 weeks long. Building on this personal experience, the mandatory part of this course was the psychological training, mainly covering knowledge on suicide and suicidal behavior, psychology of (hostage) takers, and support methods for family members or colleagues of kidnapped persons, which lasted 6 days. In particular, previously mentioned skills taught in specialized courses such as communication, de-escalation, and encounters with suicidal persons as well as PMI are typically standard components of police negotiator qualifications. As for the practical methods which convey training content, German experiences have shown the value of extensive role-playing exercises and simulation sequences, which in addition are oftentimes video recorded and assessed for feedback with the trainees.

Summing up this brief assessment of the state of the art in training law enforcement officers, we identify several significant gaps and weaknesses in the current landscape. Most importantly, finding a consensus regarding the effectiveness of the evaluation of training programs typically fails because of the unresolved issue of how effectiveness should be defined in the first place. Secondly, training programs are characterized by a great diversity (e.g., voluntariness of participation, target group, offered cash bonus payments and potential certifications) which complicates comparability. In addition, advancements in research on mental illnesses (e.g., in the connection between violence and mental disorders) must be taken into account while establishing new training concepts as to adequately address today's challenges and developments for police officers. Furthermore, we hold that such highly specialized training courses of law enforcement personnel should be designed and driven by the end-user perspective and needs to maximize proactive integration of the learning goals into the daily policing work. Without active, positive, sustainable, and intrinsic support for such training, they may have little or even no lasting impact. Finally, with this article we aim to provide a proof of concept, showing how empirically-based and end-user driven training course design in the field of mental illness awareness and response can produce up-to-date and more differentiated education concepts, that are oriented toward the trainees' own motivation and interests to increase their acceptance and compliance.

### Policing Landscape in Germany

Law enforcement in Germany is federally structured and the police force in general is separated into two main branches: the so-called uniformed police (or protection police) and the criminal investigation police. In Germany, uniformed officers, who are generally the first responders to a reported crime scene, present the major share of the police force's overall personnel. Their primary missions are threat mitigation tasks, initial on-site interrogations, and arrest or custody management. In contrast, the main tasks of the criminal investigation officers are pursuing or preventing crimes of medium or severe intensity. Their primary missions encompass advanced investigations and more detailed interrogations. Contact to mentally ill persons is frequent in both branches. In cases of self-endangerment or posing a threat to others, German police officers in the state of Baden-Wuerttemberg are legally permitted to make preliminary decisions concerning the potential necessity of medical custody for a mentally ill person, even against their own will (e.g., § 33 PolGBW^4^ and §§ 13/1, 13/3, 16/1 PsychKHGBW^5^). The final decision on a patient's admission to a medical facility, however, depends on the assessment made by psychiatric professionals. Multi-professional approaches or nationwide established training programs to improve the interaction between police officers and PMI such as in the United States, as introduced earlier do not exist in Germany yet (2). This in turn provides the optimal condition for exploring and testing methods to design training courses from scratch instead of adapting or updating existing concepts. In this sense, the German case study chosen here provides a welcoming opportunity to suggest exemplary training course components and structure to the international discourse for potential adaptation and further field testing. At the very least, our approach allows us to assess the value of incorporating end-user needs and perspectives into the training course design in this domain.

In Germany, police officers can choose between three career paths (depending on their prior education level): middle, upper and higher service. Middle service police officers are exclusively uniformed police officers whereas higher service police officers are usually tasked with personnel leadership. Starting their career in Germany, police officers receive basic education in law enforcement essentials, including mental disorders in general. Each German state has authority over their own police training and philosophies, as they all have their own legal basis for the police forces. The Police College, located within the federal state of Baden-Wuerttemberg, where the empirical survey was conducted, aims to enhance the theoretical knowledge about mental disorders, but by far not every police officer (depending on their professional career paths and simple availability of limited participant numbers) can gain access. Nevertheless, the education of police officers concerning law enforcement and their contact to PMI is located within the state's responsibility, and unfortunately its relevance can be described as optional or subsidiary in Germany.

Police officers in Baden-Wuerttemberg are legally equipped with the opportunity to label a person as mentally ill within the police-internal case management system, subject to the condition that the individuals diagnose is supported by medical certification. This procedure aims to prepare police officers with a potential warning mechanism for encounters with individuals who, based on their previous history of mental illness- have driven altercations with law enforcement personnel, and might pose a serious threat for responding officers. Among the requirements to label a person in that way, are repeated negative contacts with law enforcement personnel, previous contact to the mental health care system, and the confirmed diagnosis of a mental disorder. With such high barriers to protect against potential misuse and stigmatization (35), there is a high probability that police officers will encounter PMI without prior notification. Consequently, adequate training might significantly improve the police officers' ability to handle such challenging situations.

### General Aspects of Specialization Training in Mental Health Issues for Police Officers

We assume that a necessary condition for the successful establishment of new training programs within the police force is the trainees' acceptance. For instance, acceptance can be gained by addressing a formulated need and promising meaningful solutions to problems faced by the audience. Police forces are typically highly hierarchical organizations and the active participation and visible support by leadership positions might significantly influence the level of acceptance (30, 36). Additionally, it seems advisable to employ professionally mixed training teams that combine competencies from policing and psychology backgrounds. This might successfully foster perceived credibility and legitimacy among the audience (37, 38). A training design for police officers should also strive to utilize highly comprehensible language and learning goals that assume rather few personal experiences with mental disorders. This point was clearly influenced by the lack of education on this matter in Germany and might obviously differ in countries with a longer history of mental illness training among law enforcement personnel (39). For younger police officers and those who have just started their professional career in policing, it would be especially appropriate in our perspective to raise awareness for the likelihood and relevance concerning interactions with mentally ill people in their later policing life. All in all, we suggest to establish a training environment concerning police-PMI interactions which is accessible for all officers who are interested in the subject (30, 37, 40). Furthermore, it seems essential to design a curriculum with a central focus on feasibility and practicability. Taking in to account the daily workload of police officers (e.g., including shift work, physical and mental stress) and their mental or physical availability to participate in training sessions while on active duty, it seems beneficial to incorporate departmental requirements as well as the selection procedures of police officers who want to receive special training and be thoroughly aware of their day-to-day experiences during the training course design phase. In our exemplary training course design, we opt for a freely self-selectable and modular concept that allows adaption to shift and (arrange) schedules, as well as the general availability during working hours in order to avoid overtime. Concerning voluntary participation, research findings indicate no differences between police officers; whether self-selected or assigned to CIT-trainings regarding their empathy and psychological mindedness (41). However, some study results suggest a roughly doubled likelihood of prior exposure to mental health issues among self-selected officers, which clearly indicates a potential selection-bias if the trainings are offered exclusively on a voluntary basis (41). Still, the benefit of self-selection is validated through enhanced outcome results regarding the impact on key attitudes, skills, and adjusted behavior (42).

Based on this literature and the results of our survey, it seems advisable and feasible to create a modular and deductive curriculum design which covers general mental health education for every police officer regardless of professional assignments, and to include specialized add-on courses for subgroups of officers with a high demand for elaborated and sophisticated skillbuilding. Police officers should be given the greatest possible freedom to choose training modules, while also ensuring that the overall training components support and complement each other. Another practical problem that needs to be faced in training design is in regard to knowledge sustainability. Research indicates that in existing training concepts such as CIT officers who received training display significantly decreased knowledge about the core material, as soon as 1 month after the educational intervention. Generally, time passed was no reliable predictor of knowledge retention (43). Hence, officers might benefit more from continuous training concepts using a modular design, which allows to stretch exposure to education over a prolonged period of time (43). As a positive consequence, this might help to cover more specialized material rather than through a fixed time frame-based training design.

By the professional demands placed on police officers in their day-to-day work, pragmatism and a high intrinsic motivation to problem solving skills define policing culture (44). This means that training should always strive to be as practical and adaptable to daily policing challenges as possible. Our objective for designing the training course was to provide officers with the most effective skillset to adequately respond when in contact with mentally ill persons. Bringing these considerations together, we strongly encourage the combination of latest research results on mental health issues with simulation techniques such as role-playing scenarios, as they appear to be crucial and promising (11, 34, 36, 40, 45).

Integrating the well-established practices and positively evaluated components of existing training concepts, a central aim should be to improve communication skills, as well as non-directive active listening skills and de-escalation techniques [e.g., (31)]. Furthermore, including professionals from local mental health services or PMI themselves is highly recommended based on the positive effects of these methods as seen through CIT. A multidisciplinary approach comprising experts from police, psychiatry, and psychology to manage the theoretical fundaments and the practical training sessions is also essential, as it has been shown that police officers (due to their occupational culture) will highly validate such partnerships if they are perceived to be valuable. O'Neill and McCarthy [(44), p. 13] point out that “the tendency toward pragmatism, has actually facilitated multi-agency working in our studies. Once the officers saw the pragmatic elements of partnership work in action, this allowed them to value partnership work to the extent that previous incarnations of police culture were disturbed.” This directly supports the established positive experience from the CIT training designs that an intensive and daily work-oriented familiarization, with available community resources as alternative problem solutions for police officers while additionally, interacting with PMI are seen as highly effective (39, 46). Such a multidisciplinary collaboration should form the foundation of the training design in order to establish functional cooperation infrastructures within communities, and to further maintain a high quality of educational standards. In addition to experts and educators who are accustomed to using accessible language and presentation styles, local characteristics influencing the audience must be considered in the training design as well. This can include introducing (forensic) psychiatry or other mental health institutions with a local presence; numbers and types of police operations in urban regions; frequent suicide (attempts) and their circumstances and so on.

## Materials and Methods

### Participants and Recruitment

**Figure 1:**
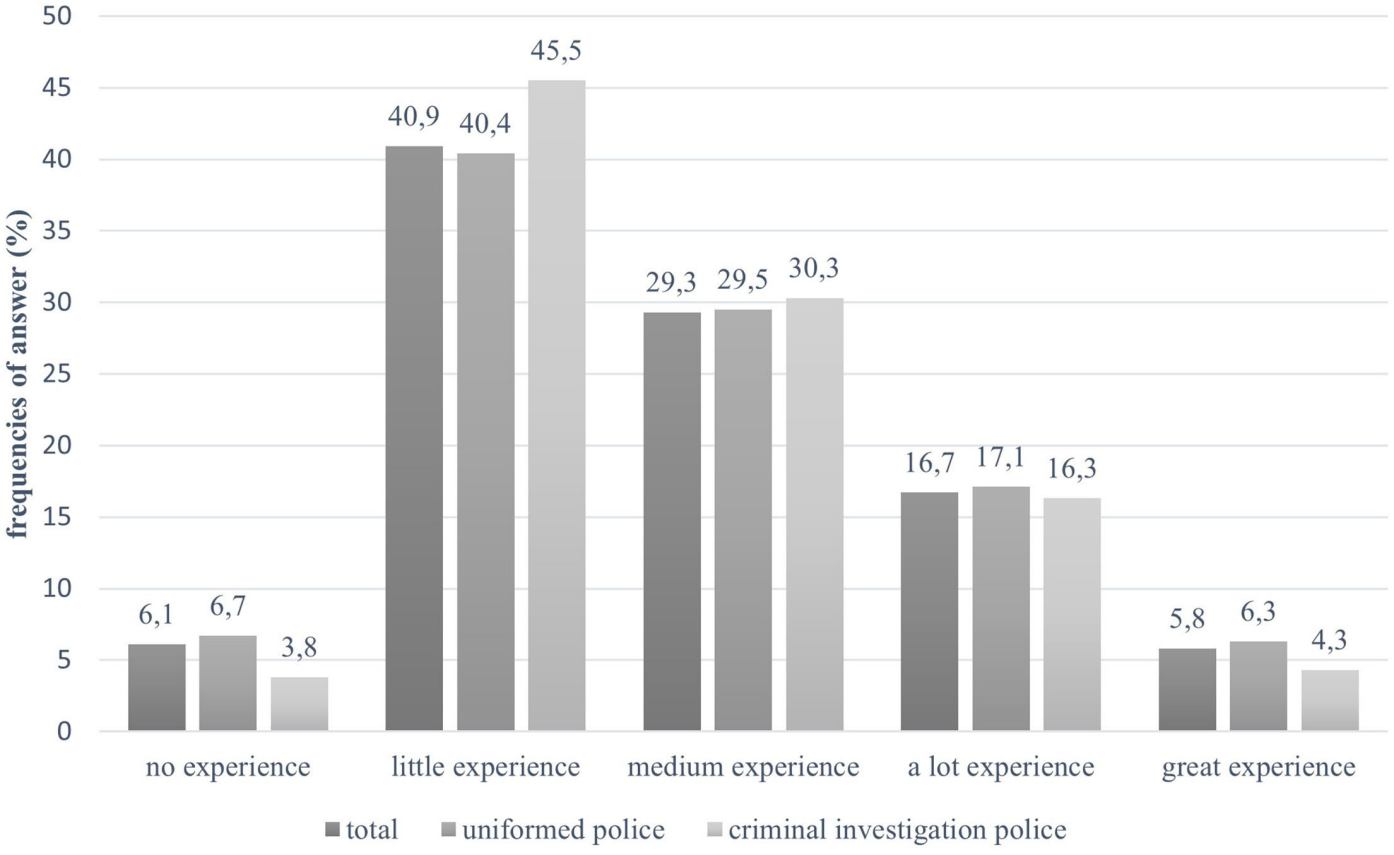
Police officers' personal experiences with mental disorders.

The exemplary training course design presented in this study in based on a questionnaire-based voluntary survey carried out among law enforcement personnel of the German federal state of Baden-Wuerttemberg (*N* = 2,228), involving the uniformed (81.1%) and the criminal investigation (18.3%) divisions of the statewide police force. Female participants accounted for 28.2% of the sample (compared to 24.8% of the overall police population) and 71.8% were male (compared to 75.2% of the overall police population). Table 1 shows the relation between the police officer population in Baden-Wuerttemberg and the study sample divided into uniformed police and criminal investigation. The study sample represents the police population in Baden-Wuerttemberg regarding career path and sex quite well. Regarding age, the study sample is however noticeably younger than the police population in general (Table 2). The participating police officers in total were active in this profession for an average of 18.2 years (*SD* = 11.8; *Min* = 0.5; *Max* = 48 years). The uniformed police officers worked 6 years less on average (17.1 years, *SD* = 11.7) than their criminal investigation colleagues (23.2 years, *SD* = 11.1). Since everyday task involve higher physical demands for uniformed police officers, the proportion of younger personnel is typically higher in that division compared to the criminal investigation branch. The comparatively high proportion of participating uniformed officers in the study sample (81.1%) may therefore explain the sample's younger age average, in the study. Concerning the police officers' personal experiences with mental illness, the largest single group among the participants reported having had limited exposure (40.9%). However, as shown in Figure 1, more than half of all officers in the sample (51.8%) report some form of personal experiences ranging from medium to significant intensity. In total, 15 police departments in Baden-Wuerttemberg were contacted, out of which 14 participated in the survey and received paper pencil questionnaires. In total, 4,455 questionnaires with a return rate of 50.01% were distributed. The estimated time for answering the questionnaire took ~20 min. Participation was anonymous, voluntary and possible during working hours. The complete and translated questionnaire used in this survey is provided in the Appendix. Each questionnaire was delivered together with a declaration of consent and data protection statement. In order to ensure anonymity, the questionnaire sheets were collected separately and returned through the departments ~6 weeks after receipt.

**Table 1:** Relation between overall police population in Baden-Wuerttemberg and study sample divided into uniformed and criminal investigation division and in regard to career path (middle, upper, higher), as well as sex (male, female).

	**Police population in Baden-Wuerttemberg (** ***N*** **=** **21,728)**		**Study sample (** ***N*** **=** **2,228)**	
	**Uniformed police**	**Criminal investigation**	**Uniformed police**	**Criminal investigation**
	**% (*N*)**	**% (*N*)**	**% (*N*)**	**% (*N*)**
Total	84.5 (18,364)	15.5 (3,364)	81.1 (1,806)	18.3 (407)
Higher service^a^	0.9 (203)	0.5 (98)	0.4 (9)	0.4 (8)
Upper service^a^	43.7 (9,501)	15.0 (3,252)	42.9 (955)	17.0 (378)
Middle service^a^	39.9 (8,660)	0.1^c^ (14)	35.9 (800)	0.4^c^ (9)
male^b^	64.1 (13,925)	11.2 (2,423)	58.7 (1,307)	12.7 (282)
female^b^	20.4 (4,439)	4.3 (941)	21.8 (485)	5.5 (123)

*^a, b^Reduced sample size because of missings*.

c *Middle service is exclusively for uniformed police officers, here officers of middle service doing an internship in criminal investigation*.

**Table 2:** Age groups in police population in Baden-Wuerttemberg and study sample in%.

	**Police population in Baden-Wuerttemberg (*N* = 21,728)**	**Study sample (*N* = 2,228)**
16–25 years	5.1	12.1
26–35 years	25.4	34.2
36–45 years	22.0	24.7
46–55 years	23.7	18.6
56–65 years	24	9.4
Prefer not to answer		0.9 (21)

### Statistical Analysis

The data was evaluated using the SPSS statistics software, version 27. The data was assessed using Chi-square-tests and Mann–Whitney *U*-tests. The Shapiro–Wilk test was used for testing data normal-distribution. Chi-square tests were used to determine whether there are statistically significant differences between sample groups e.g., between uniformed and criminal investigation police divisions, different age groups, and police officers' gender. When sample sizes were small, Fisher's exact-test was used. As a non-parametric equivalent for independent samples and because of the fact that the distributions of the groups were not equal, the Mann–Whitney *U* was preferred (instead of *t*-tests) for calculation of differences in medians. On the basis of these calculations, a training program schedule was designed, which is detailed in the following section.

## Results

The first key finding from our survey for this article's focus is that half (50.4%) of the participants expressed a strong desire to learn more about interactions with PMI, to improve their skills to adequately respond to such challenging situations and they would welcome significantly expanded training. Another 39.1% of the survey participants wished for increased networking with professionals. Regarding the establishment of supervision by mental health professionals within police force, only 13.9% of participants saw this as a favorable option. Hence, it appears to be a direct consequence of the survey to design a training course that combines multi-agency partnership building with a focus on enhancing personal skillsets required to adequately respond to specific challenges. Based on our survey results, Table 3 introduces our suggested modular training course design. While the basic module examples shown in the first row should be offered to all police officers regardless of their professional duties and specialization, the advanced modules shown in the second, third, and fourth rows should exclusively be available to certain subgroups such as uniformed police, criminal investigation police, or police officers interacting with youth and children. All modules should include role play sequences and the introduction of potential network partners. The aim of this division into basic and advanced training levels is obviously to better address the needs and interests of various groups within the law enforcement community based on their tasks and likelihood of encountering PMI in specific contexts. This fundamental training design increases the availability of basic knowledge about mental illnesses while allowing a higher degree of specialization to those officers who need it most. This might further increase acceptance and learning sustainability among the trainees.

**Table 3:** Exemplary training program.

	**All sub groups**				
Basic modules (all sub groups)^a^	*Exemplary*	*Module 1:* Mental disorders with high relevance for policing (e.g., addiction, affective disorders, schizophrenia)	*Module 2:* Learning how to respond to suicidal patients	*Module 3:* Communication techniques	*Module 4:* Mental hygiene and supervision in policing
		*Mandatory contents in all basic modules:*			
		- Introducing local mental health service professionals and networks			
		- Trialogue with (former) patients or family members			
		- Situational role play scenarios			
Advanced modules (for sub groups)		**Uniformed police**		**Criminal investigation police**	
	*Exemplary*	*Module 5:* Risks and perceived danger in contact with PMI	*Module 6:* Recognizing domestic violence and neglect	*Module 7:* Trauma sensitivity and interacting with traumatized persons	*Module 8:* Interrogation of persons being affected by sexual and/ or physical abuse
	*Exemplary*	*Module 9:* Self-protection and special characteristics in contact with PMI	*Module 10:* Successful collaboration with mental health service	*Module 11:* Interactions with children in police investigations and interrogations	*Module 12:* Indications for credibility of statements by PMI
	*Exemplary*	*Role play scenario 1:* Calming down a person with a mental disorder in a crisis situation (e.g., without medication)	*Role play scenario 2:* Transferring an intoxicated^b^ person into psychiatric care	*Role play scenario 3:* Interacting with perpetrators, victims, and witnesses in the context of interrogations and investigations	*Role play scenario 4:* Interacting methods suitable for children and adolescents
		*Network partner 1:* Patients with treated and stabilized e.g., schizophrenia or bipolar disorder^b^	*Network partner 2:* Local psychiatrists to introduce the conditions, workflow and decision-making processes in psychiatric practice	*Network partner 3:* Experts and consultants with experience in plausibility assessment during court appearance	*Network partner 4:* Local psychological-pedagogical professionals

a *Because of the fact that teaching law-related issues is better established within German police training, this curriculum draft focuses on teaching issues concerning symptom-related knowledge and adequate reactions for situations between police and PMI. In the opinion of the authors, if necessary, law-related issues (e.g., in Baden-Wuerttemberg legal statutes § 33 PolGBW and §§ 13/1, 13/3, 16/1 PsychKHGBW that regulate admission to hospital etc.) should appear on the level of the basic modules offered to all police officers*.

b *The specific diagnosis and included mental illness can vary and be adapted to the needs of the target audience*.

### Basic Modules

In addition to the theoretical background, the introduction of local mental health service professionals and networks, trialogue sequences including (former) patients or family members, and situational role-playing scenarios are essential components of this level, aiming to provide a general mental health education for police officers. The structure of the training course design also allows for the use of electronic learning platforms to cover the theoretical background and fundamentals. The modules that are outlined in detail in the following sections derive from our survey but should be seen as exemplary content that can and should be adapted to the needs and interests of the target audience.

#### Module 1: Mental Disorders With High Relevance for Policing

According to the perception of police officers who participated in our survey, certain mental disorders have been identified as highly relevant for policing work. Hence, the theoretical background module should focus on these specific disorders: the contact of police officers to patients with depression (86.3%), addiction (83.7%), and schizophrenia (78.8%), were consistently ranked highest among the survey participants. Slightly less relevant but nevertheless still very common in the eyes of the participating officers are contacts with persons suffering from borderline personality disorder (64.3%) and bipolar disorder (56.7%). Based on this perception, we measured symptom-related knowledge regarding those five mental health disorders among officers in our sample. The list was made up of 16 symptoms in total and between three and five main symptoms could be assigned to the associated disorder. Hence, a maximum score of 16 could have been reached for each symptom-related knowledge item, counting scores for including the right and excluding the wrong symptoms (*Min* = 0; *Max* = 16). Multiple answers were sometimes possible (e.g., concerning sleep disturbance). Affective disorders such as depression (*M* = 13.5) and mania (*M* = 12.8), as well as schizophrenia (*M* = 11.6) and post-traumatic stress disorder (PTSD) (*M* = 11.6) are more familiar to officers than anxiety disorders (*M* = 10.9). Combining our survey results with the state of the art in the academic discourse, as well as the experiences from another training course, we recommend that basic education be provided for police officers. This basic education should include topics relating to and discussing mental health disorders such as addiction, schizophrenia, and affective disorders since they appear to be most relevant in the perspective of the target audience. Nevertheless, we also strongly recommend the inclusion of a content-related focus on anxiety disorders and borderline personality disorders. These two disorders seem to play a common role in policing and considering our survey, criminal investigation officers have an especially high interest in the topic and need to receive training in PTSD as these officers report a much higher contact frequency (53.8%) compared to their uniformed colleagues (44.6%) (see also section Module 7: Trauma sensitivity and interacting with traumatized persons).

#### Module 2: Learning How to Respond to Suicidal Patients

The reported high frequency of suicide or attempted suicide cases is noteworthy. Three quarters of the respondents in the survey stated that they already had encountered cases of suicide (75.4%) and suicide attempts (68.9%). Learning how to adequately respond to suicidal patients therefore appears equally important for police officers as the education about mental disorders in general. In this context, training designs should include local characteristics that influence the potential case encounters for the audience (e.g., higher rates of suicide attempts in urban regions should be thematized). Since the lead author of the present study designed and accompanied a suicide-focused interaction training for German uniformed police officers in 2018, we chose to include her specific experiences here as well. During the 1-day in-house training workshop conducted by a team which consisted of a police officer and negotiation expert who experienced several suicidal (attempt) situations with PMI, the lead author taught basic statistics of the issue, facts and commonly held myths about suicide, various triggers involved, motives, and the connection to several mental illnesses. Communications techniques such as active listening skills were included in the training, as well as adjusting on-site policing measures (e.g., road closures, bystander management) and potential stressors encountered by police officers involved. Three role playing scenarios conducted by trainee actors illustrated two situations involving encounters with suicidal PMI (depression and schizophrenia), as well as an intoxicated person suffering from a personal crisis situation. Furthermore, the workshop focused on collaboration mechanisms with police negotiators, as well as emergency personnel. Finally, this specific training concept included suicide by cop incidents and legal issues. In the workshop's conclusion, the importance of specific debriefing techniques for police officers with the help of external service providers was a central part.

#### Module 3: Communication Techniques

The police officers who participated in our survey were also given the opportunity to freely indicate other challenges they experienced while interacting with mentally ill people. In order to manage and better assess these open parts of the survey, we grouped answers into categories. More than half of the police officers (56.7%) of both subgroups (uniformed and criminal investigation) experienced challenges resulting from direct contact with PMI. This category includes answers such as difficulties in “calming down,” “empathize,” “communication,” “keeping calm” and/or “building trust.” Hence, we concluded that at least one module in our training design should focus on active listening skills, the concept of empathy and empathic negotiation, techniques for building rapport, as well as situations requiring mutual respect and appreciation.

#### Module 4: Mental Hygiene and Supervision in Policing

A small yet significant percentage of the participating police officers (13.9%) suggested to establish supervision within the police force as a desirable improvement for encountering challenges resulting from contacts with PMI. Supervision can be used as a debriefing strategy after officers respond to certain potentially traumatic scenarios or preferably as a standing service for officers in general. It may be applied in one-to-one or group sessions, as well as periodically or incident focused. Well-established supervision concepts are rarely available to German police officers in our experience and are offered primarily to specialized subgroups, if at all (e.g., officers investigating cases of child pornography and abuse).

### Advanced Modules 5–8

Advanced modules should be designed to build on the previous basic modules after completion, by the target audience. These advanced components should introduce the trainees to specific methods and scenarios, such as interaction circumstances between police officers and PMI or skills to peacefully end potentially aggressive confrontations with PMI, depending on the officers' duties and responsibilities. Based on our survey results which were somewhat expected, police officers primarily encounter perpetrators of crimes (82.3%). However, only slightly less relevant are contacts with victims (72.9%). Interactions with witnesses appear to be less relevant (55.4%). Both of the latter categories of potential counterparts in police officers' interactions should not be ignored in the training design with a focus on PMI.

#### Module 5: Risks and Perceived Danger in Contacts With PMI

In the perspective of around a third of the surveyed police officers, a link between aggressive behavior shown by patients suffering from schizophrenia (34.6%) and addiction (30.4%) exists. Furthermore, the responses of almost 90% in the sample (1,983 police officers, 89.0%) indicate a potential connection between offenses involving bodily harm and addiction (35.7%), as well as patients suffering from schizophrenia (38.5%). Depression, from the perspective of police officers in our survey, is primarily linked to police operations concerning suicide and suicide attempts^6^ (25.2%). The responses of uniformed police officers concerning PMI indicate a statistically significant degree of perceived dangerousness (78.5%) and lack of predictability in behavior (67.4%) compared to their criminal investigation colleagues (68.1 and 59.3%), *p* < 0.001. Hence, this module addresses the potential knowledge gaps regarding actual threats posed by PMI, important warning signs, and mechanisms involved in increased perceptions of danger regarding mentally ill persons. The aim of this module is to improve trainees' capabilities to understand, assess, and predict the behavior of PMI instead of being influenced by stereotypes and biases that might result in stigmatization and situational escalation, driven by inadequate responses or attitudes. At this stage, officers have already been provided with the necessary basic information regarding mental disorders with high relevance for policing, as well as useful communication techniques. The most suitable target audience for this module, however, are uniformed police officers, who are typically the first responding officers at a crime scene with the mission to mitigate threats, perform initial on-site interrogations, and arrest or custody management of potential perpetrators, witnesses, and/or victims.

#### Module 6: Recognizing Domestic Violence and Neglect

The participating law enforcement officers from our survey were also asked to estimate the prevalence rates of specific types of offenses (domestic violence, physical abuse, sexual abuse, neglect, ritual abuse). In our hypothesis, these types of abuse might be associated with causing serious mental disorders. On a scale from 0 to 100%, police officers were asked to estimate the percentage of people they encountered in their day-to-day work when being exposed to these types of violence. In this context, domestic violence seems to be the most common form of violence police officers encounter (*M* = 25.7%, SD = 17.2), followed by physical abuse (*M* = 20.6%, SD = 15.7), neglect (*M* = 19.9%, SD = 16.8), sexual abuse (*M* = 13.8%, SD = 11.9), and ritual abuse (*M* = 5.4%, SD = 9.5). At this point, it is once again important to distinguish between different target groups of the training course. Neglect and domestic violence are statistically significant more often encountered by uniformed police, *p* < 0.001, while sexual and physical abuse seem to be more relevant in criminal investigations (see also section Module 8: Interrogation of persons being affected by sexual and/or physical abuse), *p* < 0.001. In consequence, we suggest including a specialized module to help uniformed police officers detect potential signs of domestic violence and neglect while entering private homes or when interacting with potential perpetrators, witnesses and/or victims.

#### Module 7: Trauma Sensitivity and Interacting With Traumatized Persons

In contrast to the previous module and according to the survey results presented in module 1, criminal investigation officers appear to have a statistically significant higher rate of contact frequency to persons suffering from PTSD (53.8%), *p* < 0.01. In addition, surveyed officers in general estimated that 14.3% of their contact persons are traumatized, with a statistically significant difference between the estimations made by uniformed (13.7%) and criminal investigation officers (16.8%), *p* < 0.001. These results fit well into the theoretical background of and expectations for the different job profiles: criminal investigation officers conduct intensive investigations and interrogations on frequent contact to perpetrators, witnesses and/or victims of potentially traumatizing (typically more serious) crimes. Consequently, we saw the need to include a specialized module on handling trauma among various types of contact persons in our course design.

#### Module 8: Interrogation of Persons Being Affected by Sexual and/or Physical Abuse

Based on our survey findings presented before, criminal investigation officers have a statistically significant higher frequency of contacts to persons who are affected by sexual and/or physical abuse. These findings should translate into a dedicated module covering these specific issues in mental health literacy training courses, such as those presented here. It is also necessary to educate participating officers on the differences in effects caused by these forms of abuse for different types of contact persons, such as perpetrators, witnesses, and/ or victims.

### Advanced Modules 9–12

#### Module 9: Self-Protection and Special Characteristics in Contact With PMI

Linked to the contents of *module 1, 3, and 5*, policing measures focused on self-protection must be critically reflected in this module. It is especially important to address uniformed police officers, who are typically the first responding officers at a crime scene. The risk of overly aggressive reactions by police officers and the use of force while encountering PMI (in the context of involuntary transfer for treatment) must be a core part of the training course and the facilitated discussion with trainees. Here, a strategy focused on involving on-site de-escalation techniques, such as negotiation, communication, and flexible tactics should be presented and practiced. The importance of physical distance, opportunities of tactical withdrawal, and the relevance of timing can be valid alternative options to pacify emotionally charged and potentially confrontational situations. Ideally, this module includes facilitation by an expert in police tactics and conflict responses, for example from Special Weapons and Tactics teams, self-defense trainers, or urban conflict specialists. As we have noted before, police culture is heavily influenced by a sense of pragmatism, which in this module clearly means and refers to the high priority of protecting themselves, fellow officers, and the public from any threats. In the context of PMI, stereotypes might result in pre-contact assumptions of severe danger to the officers' lives. This module must be designed to convey additional methods to self-protect without risking further escalation or questioning the legitimate need to avoid any unnecessary threats for the trainees. Arguably, officers will naturally opt for the self-protection methods they know best and are most familiar with. This may include the use of potentially lethal force to neutralize a threat. Hence, the aim of this module is to provide a sense of efficacy and agency in choosing from various additional de-escalation techniques in challenging situations with PMI.

#### Module 10: Successful Collaboration With Mental Health Service

It has been shown in the study by O'Neill and McCarthy (44) and supported through our survey, that police officers highly value and welcome multi-agency cooperation that helps them to improve their job performance. In our survey, 39.1% of the sample directly requested increased networking opportunities with mental health professionals. The responses of uniformed police officers indicate statistically significant more frequent challenges in collaboration with mental health services (24.4% instead of 12.7% among criminal investigation officers), *p* < 0.001. This might be due to the fact that uniformed police officers more frequently experience referral situations leading to the potential need for medical custody for a mentally ill person than their colleagues. This might include cases with high complexity, significant demands on time resources, and sometimes discrepancies with the patients' or the medical professionals' perspectives. The main goal of this module therefore is to introduce insights into professional practices and workflows within the mental health service sector, since an increased understanding of a potential collaboration partner's operations might decrease friction and practical problems of communication and referral. Survey officers for example described extremely long waiting periods or immediate discharges after transferring PMI into hospitals as highly frustrating and discouraging experiences. This may lead to reservations about mental health professionals and refraining from even attempting a collaboration. Successful collaborations are more likely when operational processes and workflows on both sides (police and mental health system) are well-known and mutual understanding has been facilitated through an exchange or constructive dialog between both professions.

#### Module 11: Interactions With Children in Police Investigations and Interrogations

Our survey results suggest that in addition to their contact with adults suffering from mental health diseases, criminal investigation officer in particular experience contact to youth (39.3%) and children (14.8%).^7^ Notwithstanding the circumstances (whether the contact person is the perpetrator, victim, or witness; mental ill or not), police officers interacting with children need to be prepared for these situations through specialized training modules. Due to the higher demands on officers to protect the well-being of children especially when they are involved in crime scenarios, a risk to cause trauma or inadequately respond to children's needs in those situations exists. Hence, this module focuses on topics around mental health issues and the needs of youth and adolescents, as well as communication techniques, in addition to the risk of heightened suggestibility while interrogations are conducted.

#### Module 12: Indications for Credibility of Statements by PMI

Criminal investigation officers in our survey describe a statistically significant higher prevalence of difficulties to assess the statements' credibility of mentally ill people during interrogations (37.6%), *p* < 0.001. Module 12 therefore addresses opportunities and limits of mentally ill persons regarding memory, plausibility, court testimony, and the risk of re-traumatization through interrogations and court appearances. Training police officers on how to distinguish between imagined statements or symptom-related episodes resulting from mental disorders and valid statements, can help to improve the effectiveness of police investigations and interrogations involving PMI. For instance, this may be achieved through better equipment and training in order to use it accordingly. In Germany, a positive process of change happened during recent years, which is, however, not completed yet; video and sound recording of interrogation situations, more standardized and sophisticated exploration methods, less use of projective procedures, fewer reporting without reality criteria, more frequent assumption of the null hypothesis (47).

### Specialized Role-Playing Scenarios and Meeting Network Partners

In addition to the suggested teaching content from the previous sections, in-depth target audience-oriented training courses should include role-paying scenarios and introduce network partners, as obvious methods to enhance police officers' learning success in preparation for encounters with PMI. Based on our survey results, the following exemplary role-playing scenarios appear to be valuable additions to the course design: (1) *Calming down a person with schizophrenia*,^8^ (2) *transferring an intoxicated person*^8^ *into psychiatric care*, (3) *interacting with perpetrators, victims, and witnesses in the context of interrogations and investigations*, (4) *interaction methods suitable for children and adolescents*. Based on the lead author's experience in police negotiator education, the training effectiveness of role-playing exercises can be increased significantly through the inclusion of debriefing and feedback components. In addition, our survey findings also support the integration of the following speaker backgrounds and network partners (who should be locally embedded in any case): (1) *patients with treated and stabilized schizophrenia*,^8^ *well-adjusted and stabilized*, (2) *local psychiatrists to introduce the conditions, workflow, and decision-making processes in psychiatric practice*, (3) *experts and consultants with experience in plausibility assessment during court appearances*, (4) *local psychological-pedagogical professionals*.

## Limitations

Self-assessment questionnaires such as the survey underlying our training course design are subjective and—in contrast to standardized observations, semi-structured interviews, or analyses of police reports—susceptible to effects of social desirability and bias. For example, working with police reports or statistics could help to objectify the number of encounter-based situations between police officers and PMI. For this, a necessary requirement must be, that the acquisition of data is standardized, which is not yet the case in Germany. The perceived danger of PMI could be one result of a biased perception in police officers, in our opinion (e.g., Halo effect, confirmation bias, fundamental attribution error). Police officers may experience especially challenging situations with PMI because of a lack of knowledge and necessary experience. Semi-structured interviews and standardized observation (especially training-related pre- and post-testing) with standardized measure values could reduce such limitations. The training concept is empirically derived. Due to the rudimentary knowledge on mental health issues that was reported by the majority of the participating police officers, the idea that their responses do not in fact reproduce the actual situation and most relevant challenges, cannot be ruled out. Internationally, policing practices as well as police education differ substantially, therefore the applicability of our results to other countries and contexts might be somewhat limited. However, since we present our training course design as an exemplary form of translating end-user perspectives into training contents (proof of concept), we are confident that the main philosophies and components are of relevance and interest to other policing environments. This is further supported by the fact that our survey includes one of the largest samples in this research field. Of course, our training course design must be understood as preliminary and theoretical in nature. Feasibility studies, empirical testing, application, and evaluation should clearly follow.

## Discussion

Scientific advances in research on mental health issues including the impact and the evaluation of mental health treatments, policing reforms, and changing mental health infrastructures clearly affect police interactions with PMI. A continuously improved understanding of these interactions as well as the awareness of the requirements for all involved actors and parties, can help to develop successful and mutually beneficial solutions to situational challenges in order to strive for the best practice approaches in this domain. Therefore, it must be pointed out that during the last few years, international collaboration projects between researchers and practitioners have focused on the development of new police officer training concepts, further improving existing ones, and expanding the overall focus of educating law enforcement personnel. In Germany, multi-professional approaches or nationwide established training programs do not exist yet (2). The mapping of existing mental health training concepts for police officers introducing this article shows a strong need for updated and more differentiated training designs. This is where our research can help urge others to reevaluate and further develop the international discourse. Our empirically-based findings from surveying German police officers about their perceptions and needs in daily policing with PMI have identified a so far largely ignored component that is essential to better inform the multidisciplinary view of this issue: the end-user perspective on mental health literacy training for law enforcement personnel. As a main result, we presented an end-user driven exemplary training course design considering the police officers' maximum degree of acceptance and perceived feasibility, to improve the incorporation of the modern state of the art between police officers and PMI. In our opinion, training program requirements should be distinguished between different police target audiences and therefore, adapt its contents to certain subgroups' individual needs; which in our case involves uniformed and criminal investigation branches. For this reason, we designed a modular, deductive, continuous, and dynamic training concept, starting with basic information for all police officers and moving to advanced topics later on for smaller subgroups. In accordance with Thomas and Watson (3) who noted: “Training is needed for everyone, but specialized training is not for all.” Similar to *TEMPO* and other training concepts (30, 37, 40), we suggest providing all police officers with basic modules and to target smaller subgroups with more specialized content. Instead of a time-based focus, we suggest a content-driven design. The theoretical inputs can be provided through electronic learning platforms. Contrary to unique training programs for police officers like CIT, we recommend a continuous training design that allows for the consistent delivery of knowledge and information. Furthermore, we recommend officers to be regularly reminded of the topic's importance and relevance, as well as to incorporate potential judicial, tactical, political, and other changes to their daily work (e.g., in the mental health system or law enforcement community). As it is the case with almost all existing training programs, we also put a strong emphasis on voluntary, practical training components (e.g., role playing) that incorporates mental health professionals, and trialogical approaches. The central focus of these trainings should be placed on de-escalation, negotiation [(48), similar to the *T3*^TM^ program], and should also include collaboration with mental health professionals as alternatives to less lethal techniques to pacify any confrontations. Even though our survey and subsequent training design are naturally specific to the German context, we are aware of the similarities it has to the education of police negotiators within the police in other countries (especially providing them with active listening skills). In addition, the increasing importance of teaching (specialized groups of) police officers about trauma sensitivity and trauma related forms of violence [e.g., neglect, domestic, sexual, and physical abuse^9^], as well as interactions with children, youth, victims, and witnesses are not challenges unique to Germany. In the United States especially, police interactions with PMI resulting in lethal and highly publicized force has directly caused significant public unrest, more violence, and community alienation from the law enforcement. Furthermore, cases of marginalization and a perceived exclusion of PMI who are at least in some countries, predominantly members of ethnic minorities, interact with larger public perceptions and allegations of systemic racism and law enforcement biases against minorities (50). In this context, a perceived lack of PMI's credibility seems to play an important role (16), which should be addressed through adequate training to ensure proper procedural justice.

The balancing act between well-equipped self-protection and proficiency in flexible tactics while encountering PMI, such as de-escalation and tactical withdrawal, are key components of the ongoing debate in many countries, including Germany. However, we strongly recommend that the current discourse and the practical application on-site be significantly improved through theoretically and practically well-trained police officers, as well as through the acceptance of police leadership; in regard to attractive, feasible, self-selected, and ongoing training concepts with clear practice orientation.

The American Psychological Association recently published a review focused on debunking myths around violence associated with mental disorders (9). It is fair to say that we are currently experiencing an encouraging phase of replacing many stereotypes and commonly held assumptions with evidence-based facts and proven methods to improve the response toward PMI. This holds great potential for professionals to adjust and update their current perspectives on this issue, mainly through better collaboration between scholars, clinicians, psychologists, contact persons, and of course law enforcement professionals.

## Data Availability Statement

The raw data supporting the conclusions of this article will be made available by the authors, without undue reservation.

## Ethics Statement

The studies involving human participants were reviewed and approved by Ulm University Medical Center. The patients/participants provided their written informed consent to participate in this study.

## Author Contributions

Both authors listed have made a substantial, direct and intellectual contribution to the work, and approved it for publication.

## Author Disclaimer

This article only represents the opinions of the authors and not necessarily that of the Ministry of the Interior, Digitalisation and Local Government of Baden-Württemberg.

## Conflict of Interest

The authors declare that the research was conducted in the absence of any commercial or financial relationships that could be construed as a potential conflict of interest.

## Publisher's Note

All claims expressed in this article are solely those of the authors and do not necessarily represent those of their affiliated organizations, or those of the publisher, the editors and the reviewers. Any product that may be evaluated in this article, or claim that may be made by its manufacturer, is not guaranteed or endorsed by the publisher.

## Footnotes

^1^Research received ethics committee IRB approval, Ulm University, Protocol Number 03/19-FSt./bal.

^2^E.g. in Waterloo, Iowa; Portland, Oregon; Albuquerque, New Mexico; in Seattle, Washington; in Akron, Ohio (27); in Colorado (28) CIT-ECHO an Extension for Community Healthcare Outcomes (29).

^3^Level explained: TEMPO 100 includes TEMPO 101–104; TEMPO 200 includes TEMPO 201–202; TEMPO 300 includes TEMPO 301; TEMPO 400 includes TEMPO 401.

^4^https://www.landesrecht-bw.de/jportal/?quelle=jlink&docid=jlr-PolGBW2021pP33&psml=bsbawueprod.psml&max=true (accessed June, 2021).

^5^https://www.landesrecht-bw.de/jportal/?quelle=jlink&docid=jlr-PsychKGBWpG3&psml=bsbawueprod.psml&max=true (accessed June, 2021).

^6^In Germany committing suicide is not a criminal offence but typically involves policing measures related to threat mitigation.

^7^According to German law (§ 1 JuSchG, https://www.gesetze-im-internet.de/juschg/__1.html) children are people who are not yet 14 years old, youth are people who are 14 years of age or older, but not yet 18 years old.

^8^The specific diagnosis and included mental illness can vary and be adapted to the needs of the target audience.

^9^For more information, see Lorey and Fegert (49).
