# Supplementary material for: Increasing Mental Health Literacy in Law Enforcement to Improve Best Practices in Policing—Introduction of an Empirically Derived, Modular, Differentiated, and End-User Driven Training Design
Source: Front Psychiatry. 2021 Aug 2;12:706587. doi: 10.3389/fpsyt.2021.706587 (PMC8365022; doi:10.3389/fpsyt.2021.706587)
Supplement: Supplementary file 1 [file Data_Sheet_1.pdf]

What role do people with mental illnesses play in everyday police life?

It is estimated that every third to fourth police contact involves a mentally ill person. Nevertheless, the topic is largely unexplored scientifically. That should change!

Your participation in this survey is an essential contribution to examine this research question - thank you very much!

As a police officer, you have contact with people who are involved in criminal offenses regularly (perpetrators, victims, witnesses, etc.). This survey is interested in the people among your contacts who show symptoms of mental illness or are mentally ill and how you experience these contacts. Therefore, we kindly ask you to answer the following questions. Please send the completed questionnaire back to me anonymously by xx/xx/xx.

Your participation is of course voluntary.

© 2019, Ulm University, LKA Baden-Wuerttemberg, author contact: katharina.lorey@gmail.com

- I. General informations -

1) First, a few questions about yourself.

Your Gender	□ man	□ diverse	
	□ woman	□ prefer not to answer	
Your group of age:	□ 16-25	□ 26-35	□ 36-45
	□ 46-55	□ 56-65	□ prefer not to answer

(other) vocational trainings ____

2) Please describe your current function / position in police work.

____

____

____

3) Please describe your career in police.

a) Currently working in:	career path:	since:
□ uniformed police	□ middle service	________
□ criminal investigation	□ upper service	________
	□ higher service	________

b) Former areas of application within police, e.g.: *state security* (for *6* years).

1. area: ____________________________ (for _____ years)
2. area: ____________________________ (for _____ years)
3. area: ____________________________ (for _____ years)

c) length of service (until today): ________ years

- II. mental disorders -

1) Test your knowledge!

Here you can see a list of symptoms (left) and 5 mental illnesses (right). Which symptoms belong to which mental illness? Write the corresponding numbers in the boxes (multiple answers possible).

1. sleep disorder
2. increased nervousness
3. flashbacks (relivining of experiences)
4. fear of specific activities
5. vertigo
6. increased restlessness
7. megalomania
8. hallucinations
9. loss of interest
10. difficulty concentrating
11. reckless/ risky behavior
12. dissociation (depersonalization/ derealization)
13. hearing voices
14. suicidal ideation
15. avoidance of being in public places
16. delusion

anxiety:

depression:

mania:

schizophrenia:

traumatization:

Personal experiences, but also genetic and / or other influences, can have an impact on both physical and mental health. These experiences and influences can manifest themselves in form of symptoms. The appearance of a certain number and combination of symptoms can indicate a mental illness.

The following section of the questionnaire examines the extent to which you have already had contact to people who showed symptoms and / or suffered from mental illnesses in your work context (e.g. during field activities, interrogations, surveys, etc.).

Important – the point is not that you give or suspect diagnoses. Your answers should refer to the people who either provided information about their illness themselves or for whom evidence was provided by third parties (relatives, doctors, etc.).

A preliminary survey with police officers from Germany has shown that these encounters include diseases such as anxiety disorder, autism, bipolar disorder (manic-depressive), "borderline" diseases (with or without self-harming behavior), burnout, depression, personality disorder, psychopathy, schizophrenia, addiction, suicide and attempted suicide and post-traumatic stress disorder (PTSD) or traumatization are well known.

1) Which mental illnesses have you personally encountered in your daily work?

____

____

____

2) What are the 3 most common mental disorders that you usually come into contact with at work?

1. ____
2. ____
3. ____

3) How many of the contacts you have in your day-to-day work are with a mentally ill person[1]? Please draw this in the diagram below.

Mentally ill persons
approx. ____ %

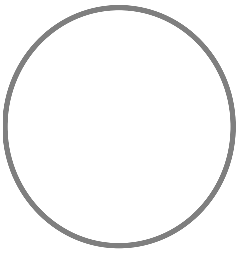

healthy persons
approx. ____ %

4) How many of the contacts that you have in your day-to-day work life are with a person, who shows mentally abnormal behavior[2]? Under consideration of this additional category, please draw this in the diagram below.

Mentally ill persons
approx. ____ %

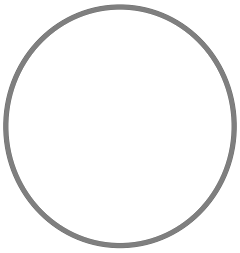

Healthy persons
approx. ____ %

Persons with mentally
abnormal behavior
approx. ____ %

[1] People who either provided information about their illness themselves or for whom evidence was provided by third parties (relatives, doctors, etc.).

[2] People with no evidence of a mental illness, but who show behavior appeared to be mentally abnormal (e.g. strange, bizarre, confused, etc.).

5) To what groups of people do you mainly have contact within your work (multiple answers possible)?

a) □ women □ men

b) □ adult □ adolescents □ children

c) □ perpetrators □ victims □ witnesses

6) Are there groups of people in which, according to your professional experience, mental illnesses accumulate?

7) In your professional experience, are specific mental illnesses related to certain offenses? If yes, please record this in the table below.

Mental disorder	offense

8) In your professional experience, are there specific mental illnesses that are associated with aggressive, unruly behavior? If yes, please write it down.

9) In your professional experience, were you in contact with a mentally ill person that (nearly) escalated? Please describe briefly (if there were several of such contacts, please estimate the number).

10) Where do you see the challenges in dealing with mentally ill people in your current occupation?

____

____

____

11) Where do you see the challenges in dealing with mentally ill people for the police in general? **Please assign 3 votes.**

- □ recognition of mental disorder
- □ dangerousness
- □ establishing contact
- □ understanding of their thoughts and actions
- □ reliability of the persons
- □ predictability of their behavior
- □ credibility of their statements
- □ press coverage
- □ personal stress
- □ collaboration with mental health service etc.
- □ ____

12) Please estimate the percentage of people you meet in your day-to-day work…

a. **…who are traumatized**?

□ 0% □ 10% □ 20% □ 30% □ 40% □ 50% □ 60% □ 70% □ 80% □ 90% □ 100%

b. …who were exposed to **physical abuse**?

□ 0% □ 10% □ 20% □ 30% □ 40% □ 50% □ 60% □ 70% □ 80% □ 90% □ 100%

c. … who were exposed to **sexual abuse**?

□ 0% □ 10% □ 20% □ 30% □ 40% □ 50% □ 60% □ 70% □ 80% □ 90% □ 100%

d. … who were exposed to **neglect**?

□ 0% □ 10% □ 20% □ 30% □ 40% □ 50% □ 60% □ 70% □ 80% □ 90% □ 100%

e. … who were exposed to **domestic violence**?

□ 0% □ 10% □ 20% □ 30% □ 40% □ 50% □ 60% □ 70% □ 80% □ 90% □ 100%

f. … who were exposed to **ritual abuse**[3]?

□ 0% □ 10% □ 20% □ 30% □ 40% □ 50% □ 60% □ 70% □ 80% □ 90% □ 100%

[3] In ritual structures of violence, sexualized, physical and / or psychological violence is systematically applied to children, adolescents, and adults through the cooperation of several perpetrators. The acts are often associated with commercial sexual exploitation and are justified or justified by an underlying ideology.

- III. change of perspective -

13) Please imagine the following scenarios:

a) On the way home, one of your acquaintances was bullied and insulted. He / she will now tell you about the circumstances. What is the likelihood you would recommend filing a complaint?

□ 0% □ 10% □ 20% □ 30% □ 40% □ 50% □ 60% □ 70% □ 80% □ 90% □ 100%

b) On the way home, one of your acquaintances was sexually harassed. He / she will now tell you about the circumstances. What is the likelihood you would recommend filing a complaint?

□ 0% □ 10% □ 20% □ 30% □ 40% □ 50% □ 60% □ 70% □ 80% □ 90% □ 100%

c) After the incident your acquaintance shows symptoms of mental illness (e.g. depression, avoidance, fear, nervousness, etc.). So far, the person has not decided to file a complaint with the police. What is the likelihood you would recommend filing a complaint?

□ 0% □ 10% □ 20% □ 30% □ 40% □ 50% □ 60% □ 70% □ 80% □ 90% □ 100%

d) Your acquaintance decided to file a complaint with the police. Today is the appointment and he/she calls you before in order to ask for advice. What will you tell him/her?

____

____

____

14) In your professional experience, what reasons do mentally ill or stressed people have for deciding against filing a complaint? **Please assign 3 votes.**

- □ fear of the consequences
- □ strains resulting from the follow-up measures (questioning, trial, etc.)
- □ the evidence is insufficient
- □ did not think about that
- □ did not feel understood
- □ „*Police doesn't believe*“
- □ „*Police doesn't do anything*“
- □ incident is statute-barred
- □ others:

____

____

____

14) We have talked a lot about the challenges in dealing with mentally ill people. In your professional experience and within police work, what is going well concerning that topic?

____

____

15) Finally, you may select **one** suggestion for the improvement of contact with mentally ill people within the police. Which one do you choose?

- □ revision of the basic training
- □ expansion of vocational training
- □ establishing a concept of supervision
- □ increased networking with professionals
- □ other:

____

- □ no necessity for improvement

16) 16) How much experience have you had in your personal environment in dealing with mentally ill people?

□ great □ a lot □ medium □ little □ no

Thank you for your participation!

Questions, suggestions, praise, criticism please email
[redacted]
